# Supplementary material for: Bridge crack detection based on improved single shot multi-box detector
Source: PLoS One. 2022 Oct 4;17(10):e0275538. doi: 10.1371/journal.pone.0275538 (PMC9531840; doi:10.1371/journal.pone.0275538)
Supplement: Attachment — Submitted filename: Response to Reviewers.docx [file pone.0275538.s001.docx]

**Original Manuscript ID:** PONE-D-22-07252

Original Article Title: Bridge Crack Detection Based on Improved Single Shot Multi-Box Detector

**To:** PLOS ONE Editor

**Re:** Response to reviewers

Dear Editor,

Thank you for allowing a resubmission of our manuscript, with an opportunity to address the reviewers’ comments.

We are uploading (a) our point-by-point response to the comments (below) (response to reviewers), (b) an updated manuscript with yellow highlighting indicating changes (*Supplementary Material for Review*), and (c) a clean updated manuscript without highlights (*Main Manuscript).*

Best regards,

<Xiaohui He> et al.

**Reviewer#1, Concern # 1:** The authors do not discuss the figures presented in the experimental results section. This is a critical issue in this section.‎

**Author response:** We gratefully appreciate for your valuable suggestions. We re-combed the writing ideas of the experimental part, focused on the specific experimental results of different networks, and analyzed the deep-seated reasons behind the various experimental results from the level of target detection mechanism. The reviewers put forward valuable opinions, which increased the article's expression logic and helped improve the overall quality of the article.

**Author action:** We revised this part of the article and highlighted it. (Line433-444 and Line452-506).

**Reviewer#1, Concern # 2:** There are lots of typos. English needs to revise again with a professional editing service. Also, the figures are not clear in some cases.

**Author response:** Thank you again for your positive comments and valuable suggestions to improve the quality of our manuscript. In view of these problems, we have updated the illustrations in the article. Unfortunately, our article has been polished by a commercial translation agency before submission, and we have further optimized the language part of the article. Although we are not very confident, we still hope it can meet your requirements. The English editing service certificate is as follows:

.

**Author action:** We improved the resolution of the pictures in the article to 300dpi. The modified pictures are as follows:

**FIG 1.** **Structure of SSD.**

**FIG 2.** **Structure of ISSD.**

**FIG 3. Three typical cases of convolution kernel shifting of conventional convolution.** Group A is the conventional distribution, Group B is the distribution after arbitrary migration, Group C is the distribution after scaling transformation and Group D is the distribution after rotational transformation.

**FIG 4. Schematic diagram of depth separable convolution.**

**FIG 5. Schematic diagram of the inception structure**. Group A represents the structure of the original inception and Group B represents the structure of the improved inception.

FIG 6. Structure of the Feature recalibration module.

**FIG 7.** **Curves of training loss with different learning epochs.**

**FIG 8. Comparison of output results at different epochs.** The green boxes locate the missing detection parts of the detection results and the red boxes locate the false detection parts of the detection results.

**FIG 9.** **Visualization of detection results of compared networks on the WCD dataset.** From left to right: original images, GT, FCN, U-Net, CrackDAFNet, ISSD.

**FIG 10.** **The visualization of detection results of compared networks on special cases.** From left to right: original images, GT, FCN, U-Net, CrackDFANet, ISSD.

**FIG 11.** **Precision-recall curves of composed networks on WCD dataset.**

**Reviewer#1, Concern # 3:** Mention the limitations and future works of the developed system elaborately.

**Author response:** We gratefully appreciate for your valuable suggestions. In the conclusion of the article, we summarize the advantages and disadvantages of the network proposed in this paper, and formulate the direction of the next work.

**Author action:** We updated the conclusion of the article to make the structure of the full text more complete. (Line 518-526).

**Reviewer#1, Concern # 4:** Several techniques have been described in the Introduction section. How do the authors outperform each of these reviewed systems? A clear statement is needed to highlight the contribution (use the table to discuss each method).

**Author response:** Special thanks to you for your good comments. This paper designs a bridge crack detection network based on the deep learning theory. The work of this paper is only a part of the bridge crack detection project. The advantage of the detection network based on computer vision is its learnability. The network can learn the relevant knowledge of crack detection from the data samples and can detect the bridge crack accurately and quickly by relying on the mighty computing power of the convolution neural network. Compared with other traditional detection technologies, crack detection methods mainly rely on artificial vision inspection or instrument signal characteristic analysis. The detection accuracy and speed lag behind the detection network designed in this paper. However, bridge crack detection is a complex engineering project. The whole project includes many hardware, software, and corresponding manual operations. The design of bridge crack in this paper is a systematic engineering project, so it is impossible to compare with other detection technologies from the overall perspective of bridge crack detection engineering.

**Author action:** We revised the description of highlighting the traditional detection methods and the advantages and disadvantages of the network designed in this paper. (Line39-41, Line51-54 and Line 62-82).

**Reviewer#1, Concern # 5:** Discuss the stability of the system in terms of complexity.

**Author response:** Thanks again to the reviewer, we are also aware of this problem. Network parameter quantity is an index to evaluate the complexity of a network system based on deep learning. We add this part to the article and combine it with the network's accuracy and reasoning speed to analyze the network's stability from the perspective of complexity. In addition to the content shown in the manuscript itself, we tested it on different training and test sets and different computing platforms, and the test results did not change significantly. Therefore, our network has stable performance under low computing power dependence after sufficient training. Due to the article's length, we don't think putting these details in the manuscript is necessary.

**Author action:** We added this part in the manuscript. (Line 452-464 and Table 5).

**Reviewer#1, Concern # 6:** Detailed evaluation of how the proposed algorithm performs and the varying parameters must be provided. You only provided a learning rate.

**Author response:** Special thanks to you for your good comments. Network parameters and training mode play a vital role in the final performance of the network. Combined with the characteristics of bridge crack detection, we show more detailed information on the network training process in the article and comprehensively analyze the network's performance in different gradient descent modes and different iterative stages.

**Author action:** We show more details of the network training process in the article, analyze the speed and performance of network training under different gradient descent modes, and compare the network's performance under different iterative stages on this basis. (Line386-412 and Fig.8).

**Reviewer#1, Concern # 7:** All the figures must be in 300dpi.

**Author response:** Thank you again for your positive comments and valuable suggestions to improve the quality of our manuscript. In view of these problems, we have updated the figures in the article.

**Author action:** The fonts in our picture has been replaced, and the resolution of the picture has been increased to 300ppi.

**Reviewer#1, Concern # 8:** Parametric settings need to discuss. How you select the parameters in your model?

**Author response:** Special thanks to you for your good comments. This question has been partially answered in question 6.

**Author action:** We show more details of the network training process in the article, analyze the speed and performance of network training under different gradient descent modes, and compare the network's performance under different iterative stages on this basis. (Line386-412 and Fig.8).

**Reviewer#1, Concern #9:**  The conclusion is confusing. You need to change it. Should be according to the body of the manuscript. You must write some findings in the conclusion section.

**Author response:** It is true as Reviewer suggested that conclusion in the article is not clear in expression. We rewrote the conclusion chapter, focusing on the overview of the work done and the results achieved in this paper. In addition, according to the problems found in the experimental process, we analyzed the shortcomings of the work done in this paper and made a direction for future work.

**Author action:** We updated the manuscript by adding the description of this part in the article. (Line 518-526).

**Reviewer#1, Concern # 10:** All the figures are not cited properly in the text.

**Author response:** Thank you again for your positive comments and valuable suggestions to improve the quality of our manuscript. Before submitting the manuscript, we have arranged the article in strict accordance with the requirements of the journal. We will continue to communicate with the editor to determine the citation format of the pictures in the article.

**Reviewer#2, Concern # 1:** In line 52, what subjective factors are in consideration should be indicated and elaborated.

**Author response:** Special thanks to you for your good comments. The core problem of the effect of the detection method based on manual design features is the quality of manual design features, which are usually completed manually, and subjective factors are inevitably introduced in the feature design process.

**Author action:** We updated the manuscript by modifying the description of this part. (Line 51-54)

**Reviewer#2, Concern # 2:**  In lines 59, 61, and 64, the complex environmental noise should be elaborated in terms of its nature and in terms of its effect on detection.

**Author response:** Thank you again for your positive comments and valuable suggestions to improve the quality of our manuscript. In view of these problems, we have updated the illustrations in the article.

**Author action:** We updated the manuscript by adding the description of this part. (Line62-68)

**Reviewer#2, Concern # 3:** There are inconsistencies between SSD layer names in lines 95-100 and SSD layer names in Fig 1. In [21], there is a Conv6 layer as also indicated in line 96, however Figure 1 does not have it.

**Author response:** Thanks again to the reviewer, we are also aware of this problem. We updated the content of Figure 1 to conform to the description of this part in the article.

**Author action:** We have updated the content of Figure 1. The modified Fig. 1 is as follows.

original image

Updated image

**Reviewer#2, Concern # 4:** In general, Fig 2 is not digestible as a whole. Please give data structure information (i.e., dimensions) about colored boxes. There are color inconsistencies between figure legend and the figure. It is not evident where the composite structure sits in the architecture nor there is a reference about it in the text. Please carefully review the figure to make it easy to comprehend and more connected to the text.

**Author response:** Special thanks to you for your good comments. We have replaced Fig2. We revised figure 2, added the corresponding parameters of the feature layer, adjusted the corresponding relationship between the legend and the legend color, and marked the position of the composite structure in the network. The composite structure of this paper is designed by ourselves. There is no relevant reference. We explain it in the corresponding position in the article.

**Author action:** We added a description of Figure 2 in the article (Line129-136) The modified Fig.2 is as follows.

original image

Updated image

**Reviewer#2, Concern # 5:** References and the text are not consistent:[24] is not work of Zhao et al. Lu et al.'s work [25] is not about bridge crack detection as asserted by the authors. Feng et al.'s wok [26] is not about bridge crack detection. However, on can stress that they are correlated problems. Just align the previous work and your statements.

**Author response:** We gratefully appreciate for your valuable suggestions. Bridge cracks and pavement cracks are both concrete surface cracks. From the perspective of network engineering applications, we expect that the network designed in this paper is not limited to bridge crack detection, so we refer to many similar cracks detection research.

**Author action:** We have revised the relevant parts of the article to strengthen the preciseness of the expression of the article. (Line 112-121 and Line 579-584)

**Reviewer#2, Concern # 6:** [28] is not about deformable convolutions. Please update all the references in the manuscript and make them consistent to the text. The manuscript is not scrutable with that state.

**Author response:** We are very sorry for our negligence in this manuscript, we reviewed the address of reference [28] and revised the address in the manuscript. In addition, we reorganized all references and revised 6 references.

**Author action:** Once again, we reorganized all the reference and revised 6 references in this manuscript. (Line566-567, line579-584 and line588-597)

**Reviewer#2, Concern # 7:** In Section 3.1.1, deformation convolution is illustrated however it does not give any credit to the its original inventors: https://arxiv.org/pdf/1703.06211.pdf Please cite properly. ([27] is not that paper.)

**Author response:** We gratefully appreciate for your valuable suggestions. We reviewed the address of reference [27] and revised the address in the manuscript.

**Author action:** The revised address of reference [27] in the manuscript is highlighted. (Line 588-591)

**Reviewer#2, Concern # 8:** In Section 3.1.2, in line 160, the reference [29] is not about depth separable convolution. The DOI in [29], doi:10.4271/2014-01-0975, is not a paper titled Xception. It is titled as Fatigue Behavior of Stainless-Steel Sheet Specimens at Extremely High Temperatures.

**Author response:** We gratefully appreciate for your valuable suggestions. We reviewed the address of reference [29] and revised the address in the manuscript.

**Author action:** The revised address of reference [29] in the manuscript is highlighted. (Line 595-598)

**Reviewer#2, Concern # 9:** WCD dataset does not have any reference, thus not accessible.
NOTE: This manuscript has too many fundamental errors. Please carefully review the paper and then I will be able to read it with a mind in peace.

**Author response:** Thanks again to the reviewer, we are also aware of this problem. We didn't consider this problem in the process of writing the manuscript. WCD data set is a new data set obtained after merging the existing public data sets (SDNET and CCIC), which is not described too much in the first draft. After the reviewer's reminder, we added this part to the manuscript. At the same time, we checked the manuscript more carefully to ensure that there were no basic errors.

**Author action:** We added this part to the manuscript and carefully checked the full text of the manuscript. (Line312-317)
