# Supplementary material for: Bridge crack detection based on improved single shot multi-box detector
Source: PLoS One. 2022 Oct 4;17(10):e0275538. doi: 10.1371/journal.pone.0275538 (PMC9531840; doi:10.1371/journal.pone.0275538)
Supplement: Attachment — Submitted filename: Response to Reviewers.docx [file pone.0275538.s002.docx]

**Original Manuscript ID:** PONE-D-22-07252R1

Original Article Title: Bridge Crack Detection Based on Improved Single Shot Multi-Box Detector

**To:** PLOS ONE Editor

**Re:** Response to reviewers

Dear Editor,

Thank you for allowing a resubmission of our manuscript, with an opportunity to address the reviewers’ comments.

We are uploading (a) our point-by-point response to the comments (below) (response to reviewers), (b) an updated manuscript with yellow highlighting indicating changes (*Supplementary Material for Review*), and (c) a clean updated manuscript without highlights (*Main Manuscript).*

Best regards,

<Xiaohui He> et al.

**Reviewer#1, Concern # 1:** Discuss the stability of the system in terms of complexity.‎

**Author response:** We gratefully appreciate for your valuable suggestions. In the experimental part of the manuscript, we added experiments on network computing efficiency and computing power cost, and comprehensively analyzed the relationship between network complexity, computing power consumption and network performance.

**Author action:** We revised this part of the article and highlighted it. (Line448-466, Table 5 and Table 6).

**Reviewer#2, Concern # 1:** In the lines 234-243, the authors discuss an improved version of the original inception module and illustrates the improved version at Fig5b. Inception-v3 is presented in the “Rethinking the Inception Architecture for Computer Vision” of the Szegedy et. al.’s work. The authors should elaborate their improvement of Fig5b over the Fig5 of Szegedy’s Inception-v3 work. If that network is implied, please reference it. In the Fig.5b, the network concatenates onto “previous layer”, it should be checked.

**Author response:** Special thanks to you for your good comments. The expression of the relevant contents of the manuscript is not clear and does not highlight the key points. We have modified the relevant contents of the manuscript to make the contents of the manuscript more rigorous.

**Author action:** We updated the manuscript by modifying the description of this part. (Line 222-238)

**Reviewer#2, Concern # 2:**  In section 3.1.3, the marriage of depth wise separable convolutions and deformations convolutions proposed. However, its inner structure and mechanism is not explained and illustrated in a figure other then a box in the Fig2. The mechanism of the proposed combination network should be illustrated and explained other than or additional to lines 212-219.

**Author response:** Thank you again for your positive comments and valuable suggestions to improve the quality of our manuscript. In view of these problems, we have added the structure diagram of relevant parts.

**Author action:** We updated the manuscript by adding the structure diagram of this part.

**FIG 5. Schematic diagram of the depth separable deformable convolution**.

**Reviewer#2, Concern # 3:** The line 285 reads L(x,c,l,g) while eq. (14) doesn’t have such a term.

**Author response:** Thanks for your careful checks. We are sorry for our carelessness. Based on your comments, we have made the corrections to make the unit harmonized within the whole manuscript.

**Author action:** We have checked the manuscript again to ensure its quality.

**Reviewer#2, Concern # 4:**  In Section 4, authors compare performance of their proposed network with a non-SSD based networks such as CrackDFA, however, with a quick search, there is another proposed work of Xu et. al. titled “Automatic Bridge Crack Detection Using a Convolutional Neural Network” which is also non-SSD based method however based on the depth wise separable convolutions. Authors are urged to add more literature on the problem of “bridge crack detection” with other NNs not just SSD and add comparisons also on such papers.

**Author response:** Special thanks to you for your good comments. We added more comparison networks in the comparison experiment, and carried out the comparison experiment under the same experimental platform and experimental settings, which made the performance comparison objects of our network more diverse, not limited to SSD based networks, and increased the persuasion of the manuscript.

**Author action:** We updated the manuscript by modifying the description of this part. (Line 303-306, 448-466 and 471-512, Table 5, Table 6, Fig. 10 and Fig. 11)
