# Supplementary material for: Bridge crack detection based on improved single shot multi-box detector
Source: PLoS One. 2022 Oct 4;17(10):e0275538. doi: 10.1371/journal.pone.0275538 (PMC9531840; doi:10.1371/journal.pone.0275538)
Supplement: Attachment — Submitted filename: Response to Reviewers.docx [file pone.0275538.s003.docx]

**Original Manuscript ID:** PONE-D-22-07252R1

Original Article Title: Bridge Crack Detection Based on Improved Single Shot Multi-Box Detector

**To:** PLOS ONE Editor

**Re:** Response to reviewers

Dear Editor,

Thank you for allowing a resubmission of our manuscript, with an opportunity to address the reviewers’ comments.

We are uploading (a) our point-by-point response to the comments (below) (response to reviewers), (b) an updated manuscript with yellow highlighting indicating changes (*Supplementary Material for Review*), and (c) a clean updated manuscript without highlights (*Main Manuscript).*

Best regards,

<Ziting Zhu> et al.

**Reviewer#2, Concern # 1:** Thank you for addressing most of my comments. I have concern about one comment I did in the second revision request. I believe I couldn't be clear about it. I originally mentioned about Szegedy’s Inception-v3 work. There are three models of interest that are portrayed in three figures.
 Figure X: Figure 2(b) of Going deeper with convolutions (https://arxiv.org/pdf/1409.4842.pdf).
 Figure Y: Figure 5 of Rethinking the Inception Architecture for Computer Vision (https://arxiv.org/pdf/1512.00567.pdf).
 Figure Z: Figure 6(b) of this manuscript (previously Figure 5(b))
 Figure X is the original Inception module of Szegedy et. al.'s work, that is authors of this manuscript also indicate an improved version over the Figure X.
 Figure Y is an improved version of Figure X which is also proposed by Szegedy et. al.
 Figure Z is the improved version of Figure X that authors proposes.
 In lines 222-238, the authors beautifully explained how this proposed network works and why those layers are chosen. However, I urge the authors to comment on the improvement of the model in Figure Z over the model in Figure Y. In other words, models in Figure Y and Z should be compared.

**Author response:** Special thanks to you for your good comments. The expression of the relevant contents of the manuscript is not clear and does not highlight the key points. We have modified the relevant contents of the manuscript to make the contents of the manuscript more rigorous.

**Author action:** We updated the manuscript by modifying the description of this part. (Line 239-243)
